# The Emerging Scenario of Ferroptosis in Pancreatic Cancer Tumorigenesis and Treatment

**DOI:** 10.3390/ijms252413334

**Published:** 2024-12-12

**Authors:** Hao Lyu, Jinghua Kong, Jiasi Chen, Rui Zhang, Shuai Xiao, Dong Guo, Qi Zhang, Xing-Zhen Chen, Jingfeng Tang, Cefan Zhou

**Affiliations:** 1 National “111” Center for Cellular Regulation and Molecular Pharmaceutics, Key Laboratory of Fermentation Engineering (Ministry of Education), Hubei University of Technology, Wuhan 430068, China; 2 Cooperative Innovation Center of Industrial Fermentation (Ministry of Education & Hubei Province), Hubei Key Laboratory of Industrial Microbiology, Hubei University of Technology, Wuhan 430068, China; 3 Membrane Protein Disease Research Group, Department of Physiology, Faculty of Medicine and Dentistry, University of Alberta, Edmonton, AB T6G 2R3, Canada

**Keywords:** pancreatic cancer, ferroptosis, tumorigenesis, drug resistance, immunotherapy

## Abstract

Pancreatic cancer remains one of the most lethal forms of cancer. Currently, there is a lack of effective drug treatments for pancreatic cancer. However, as a newly discovered form of non-apoptotic cell death, ferroptosis has garnered increasing attention in relation to pancreatic cancer. Understanding the role of ferroptosis in the tumorigenesis and treatment of pancreatic cancer may enable more effective clinical trials and treatments for pancreatic cancer and may minimize side effects or restrict the emergence of drug resistance. In this review, we summarize the current knowledge regarding the process and underlying mechanisms of ferroptosis, as well as its dual role in both promoting tumorigenesis and facilitating treatment strategies for pancreatic cancer. Additionally, how ferroptosis is implicated in the development of pancreatitis and insulin resistance, indicating that ferroptosis may play an important role in the risk of pancreatitis- and insulin-resistance-related pancreatic cancers, is also addressed.

## 1. Introduction

Pancreatic cancer (PC) is a highly malignant tumor of the digestive system and is the third leading cause of death for men and women in the United States, with a 5-year survival rate of less than 11% [1]. It is estimated that PC will become the second leading cause of cancer-related deaths by 2030 [2]. Pancreatic cancer includes a variety of different classes of malignant tumors originating from the exocrine or endocrine tissues of the pancreas. The most common is pancreatic adenocarcinoma, accounting for approximately 85% of cases [3]. Currently, the screening methods for pancreatic cancer are relatively limited. The majority of patients do not have a single attributable risk factor, further complicating the diagnosis; localized disease is mostly asymptomatic or associated with ambiguous symptoms, and the anatomical location of the pancreas is inaccessible for routine clinical monitoring [4]. For high-risk populations such as individuals with a family history or genetic mutations, gene mutations significantly related to the pathogenesis of pancreatic cancer can be detected, such as *KRAS*, *TP53*, *SMAD4*, *BRCA1*, *BRCA2*, *CDKN2A*, and *PALB2* [5,6,7]. At present, the imagiological examination for the early diagnosis of pancreatic cancer is not sufficiently sensitive [8,9,10,11]. A high level of serum carbohydrate antigen 19-9 (CA19-9) characterizes a poor prognosis for PC but has been shown repeatedly to be inaccurate and have poor diagnostic performance [12,13].

Since ferroptosis was coined in 2012, the number of relevant studies on ferroptosis has grown at an exponential rate. Ferroptosis is an iron-dependent, non-apoptotic programmed cell death mode and is characterized by an iron-dependent peroxidation reaction of phospholipids [14]. The dysfunction of ferroptosis has been associated with many physiological functions, such as tumor suppression, immune function, development, and aging [15]. Reactive oxygen species (ROS), polyunsaturated fatty acid phospholipids, and transition metal iron play a crucial role in mediating membrane damage and subsequent ferroptosis [16]. Studies have shown that histone modification, DNA modification, RNA modification, non-coding RNA, transcriptional regulation or post-translational modification, and other forms of epigenetic or non-epigenetic regulation of ferroptosis are ultimately achieved through the above three aspects [17,18,19,20,21,22,23,24,25,26]. External and internal pathways play a crucial role in the occurrence of ferroptosis. The inhibition of cell membrane transport systems, such as cystine/glutamate reverse transporters (system Xc^−^) or the activation of iron transporters, is an extrinsic pathway for the activation of ferroptosis [27]. Inhibiting the activity of system Xc^−^ prevents cystine absorption, thereby reducing the synthesis of glutathione (GSH) [28,29]. Moreover, iron-metabolism-related proteins, such as ferritin light chain (FTL), ferritin heavy chain 1 (FTH1), and transferrin receptor (TFRC), orchestrate iron storage and uptake, affecting iron homeostasis and regulating ferroptosis [30,31]. Endogenous-pathway-induced ferroptosis can be achieved by blocking intracellular antioxidant enzymes, such as glutathione peroxidase 4 (GPX4) [32]. GPX4 is one of the most efficient enzymes for clearing membrane lipid peroxides [33], and the inhibition of its activity can lead to the accumulation of lipid peroxides, resulting in ferroptosis [34].

The tumorigenesis, progression, and treatment of pancreatic cancer have always posed a clinical challenge due to pancreatic cancer’s association with various risk factors such as smoking, family history of pancreatic cancer, pancreatitis, obesity, and type 2 diabetes (T2DM) [10]. Although the discovery of ferroptosis came relatively late, recent research has indicated a close correlation between ferroptosis and pancreatic cancer [35,36,37,38]. This article provides an overview of the mechanism underlying ferroptosis and its current perspectives in terms of pancreatic cancer progression and treatment strategies for drug resistance in this context as well as pancreatitis- and insulin-resistance-associated pancreatic cancer. It offers valuable insights into comprehending the intricate role played by ferroptosis in pancreatic cancer.

## 2. The Resistance System in Ferroptosis

**Figure 1:**
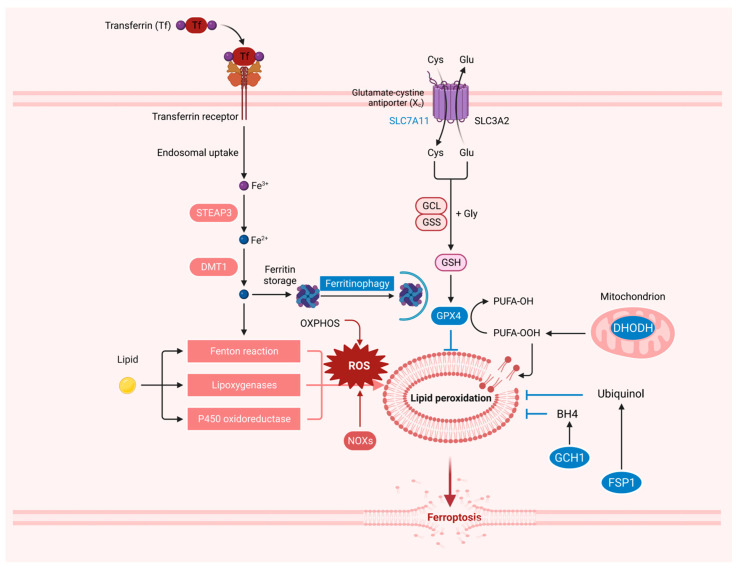
Resistance system of ferroptosis. Ferroptosis is a form of non-apoptotic cell death caused by iron-dependent membrane lipid peroxidation. Cells have evolved at least four systems inhibiting ferroptosis with different subcellular localizations to decrease lipid peroxides. The system xc^−^-GSH-GPX4 axis can collaborate with the FSP1-CoQH2 axis on the plasma membrane and can also cooperate with the DHODH-CoQH2 axis on the mitochondrial membrane. The GCH1-BH4-DHFR axis also acts as a potent cellular antioxidant providing protection from ferroptosis in the absence of GPX4. Created with BioRender.com.

Ferroptosis is a new type of regulatory cell death (RCD). During the past decade, the regulatory mechanism of ferroptosis has been gradually unraveled (Figure 1). Phospholipid hydroperoxides (PLOOHs), a lipid-based form of ROS, have been identified as the executors of ferroptosis. To cope with ROS-induced oxidative damage, cells have developed effective antioxidant defense systems [39,40].

System x_c_^−^-GSH-GPX4 axis. Most cancer cells obtain intracellular cysteine primarily through system x_c_^−^-mediated uptake of cystine, followed by cystine reduction to cysteine in the cytoplasm [41]. Cysteine further mediates the synthesis of GSH [42]. Solute carrier family 7 member 11 (SLC7A11, also known as xCT) is a subunit of system x_c_^−^ [43]. Treatment with low-cystine medium or erastin blocked SLC7A11-mediated cystine transport [44]. GSH is a cofactor of GPXs and is essential for GPX activity. GPX4 is the only intracellular enzyme that reduces lipid peroxides to lipid alcohols, thus acting as a central inhibitor of ferroptosis in cancer cells [34]. GPX4 inhibition can induce lipid peroxidation and subsequently ferroptosis in vitro and in vivo [34,45,46,47]. GPX4 consists of three isoforms with different subcellular localizations, namely cytoplasmic, mitochondrial, and nuclear GPX4 [48]. Recent studies have shown that cytoplasmic and mitochondrial GPX4 are important in ferroptosis defense in different subcellular compartments [40]. The potential role of nuclear GPX4 in ferroptosis regulation remains to be studied [47]. However, some cancer cell lines are still resistant to ferroptosis upon GPX4 inactivation, indicating the existence of alternative mechanisms.

FSP1-CoQH2 axis. In recent years, studies have found that in addition to the GPX4-dependent ferroptosis resistance mechanism, several other ferroptosis resistance systems do not depend on GPX4. Ferroptosis inhibitory protein 1 (FSP1, also known as AIFM2), as an NAD(P)H-dependent oxidoreductase, mediates the reduction of ubiquinone (also known as coenzyme Q or CoQ) to ubiquinol (CoQH2) [49,50,51]. CoQ10 often acts as an electron transport carrier on the mitochondrial respiratory chain, thereby participating in the production of cell energy. CoQH2 is also involved in recruiting lipid peroxyl radicals to inhibit lipid peroxidation and ultimately inhibit ferroptosis [47]. Recent studies have found that Ferroptosis suppressor protein 1 (FSP1, also known as AIFM2) produces non-mitochondrial-derived CoQH2 pools that inhibit ferroptosis by inhibiting lipid peroxidation through genome-wide screening [51]. Except for the inner mitochondrial membrane, the role of CoQ is largely undefined in organelles [52,53]. Therefore, whether CoQ in other membrane structures can act on lipid peroxidation in ferroptosis remains to be further studied.

GCH1-BH4-DHFR axis. Recent studies have found that guanosine triphosphate (GTP) cyclohydrolase-1 (GCH1) acts as a potent cellular antioxidant providing protection from ferroptosis in the absence of GPX4. GCH1, which is the rate-limiting step in the production of the metabolite tetrahydrobiopterin (BH4), inhibits ferroptosis through its BH4 and dihydrobiopterin (BH2) [54,55]. Dihydrofolate reductase (DHFR) uses NADP+/NADPH as a cofactor to mediate BH4 regeneration from oxidized pterin BH2. In GCH1 knockout cells, BH4 supplementation directly reversed DHFR inhibition-induced ferroptosis [56]. BH4 has a selectively protective effect on membrane phospholipids containing two polyunsaturated fatty acid (PUFA) tails from oxidative degradation, which may involve a dual mechanism: serving as a direct antioxidant and participating in the synthesis of ubiquinones [56,57]. The GCH1-BH4-DHFR system also directly inhibits ferroptosis by inhibiting lipid peroxidation, but the specific subcellular localization remains unclear. In addition, although studies have shown that GCH1 deficiency in mice leads to bradycardia in the second trimester of pregnancy and embryonic death, the mechanism of GCH1 in protecting tissues and organs from ferroptosis remains to be elucidated [58].

DHODH-CoQH_2_ axis. Recent studies have found another ferroptosis resistance system present in mitochondria [40]. Dihydroacid dehydrogenase (DHODH) is located on the outer surface of the inner mitochondrial membrane (IMM), which is involved in pyrimidine synthesis and reduces CoQ to CoQH2 [59]. DHODH inhibits ferroptosis by reducing CoQ to CoQH2 in mitochondria when mGPX4 is acutely inactivated in mitochondria. Mitochondrial mGPX4 and DHODH can collaborate to enhance the inhibition of mitochondrial lipid peroxidation, but cytoplasmic GPX4 and FSP1 cannot [47]. This indicates that the same protein plays different roles in different subcellular localizations, and it is of great significance to study ferroptosis with different subcellular localizations.

## 3. The Iron, ROS, and Lipid Metabolism in Ferroptosis

**Figure 2:**
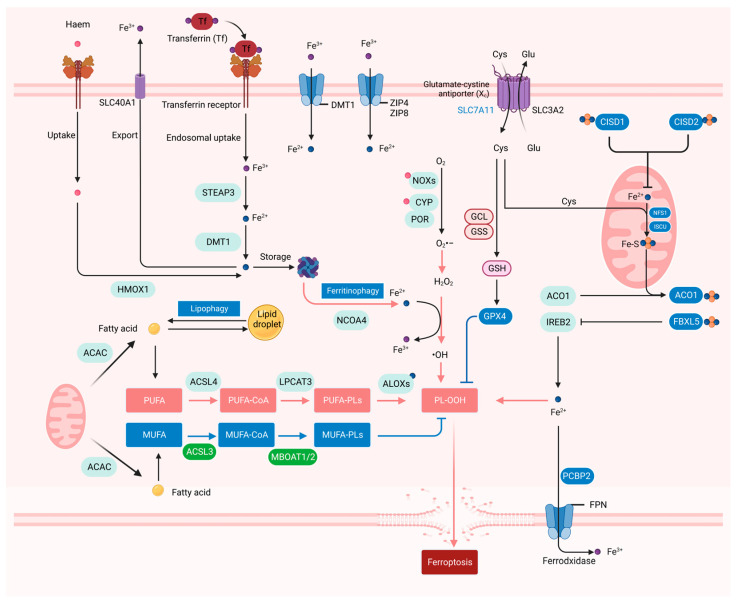
The iron, ROS, and lipid metabolism in ferroptosis. Free iron enters or exits cells through different transport pathways. Free iron mediates the production of ROS through the Fenton reaction to promote lipid oxidation and is involved in the ferroptosis process as cofactors of iron-dependent enzymes; the main sources of cellular ROS production are mETC and NOXs on the cell membrane; lipid droplets are the place where neutral lipids are stored in cells. Lipophagy induces autophagic degradation of lipid droplets and promotes the release of free fatty acid. Oxygenases, such as ALOX and POR, catalyze the oxidation of PUFAs and activate lipid peroxidation. Created with BioRender.com.

Mechanistically, ferroptosis is a form of non-apoptotic cell death caused by iron-dependent membrane lipid peroxidation. Although other RCDs can also be triggered by ROS overload, ferroptosis is induced by significant changes in the imbalance of iron-dependent ROS homeostasis and lipid metabolism. Changes in genes related to iron accumulation, GSH depletion, and lipid metabolism have been reported to be involved in ferroptosis (Figure 2).

Iron metabolism. The role of iron as the driver of membrane lipid peroxidation is mainly reflected in iron-dependent enzymes such as arachidonate lipoxygenases (ALOXs) and cytochrome P450 oxidoreductase (POR), which are involved in lipid peroxidation, and unstable iron pools that promote the Fenton reaction [60,61]. ALOX15 usually uses free PUFAs as substrates to oxidize polyunsaturated phosphatidylethanolamine (PE). Recent studies have reported that phosphatidylethanolamine-binding protein 1 (PEBP1), a scaffold protein inhibitor of protein kinase cascade, forms complexes with two ALOX15 subtypes, 15LO1 and 15LO2, and changes their substrate activity to produce hydroperoxy-PE. PEBP1-dependent ferroptosis has been shown to play an important role in airway epithelial cells in asthma, renal epithelial cells in renal failure, and cortical and hippocampal neurons in brain trauma [62]. ALOX12, another member of the ALOX family, is required for p53-dependent ferroptosis and is not required for erastin- or GPX4-inhibitor-induced ferroptosis. Mechanistically, p53 can indirectly inactivate the function of ALOX12 by inhibiting the transcription of SLC7A11 to release the lipoxygenase ALOX12 from the inactive ALOX12-SLC7A11 complex [63]. In addition, p53 can also induce ferroptosis independent of the SLC7A11/GPX4 axis by inducing spermidine/spermine N1-acetyltransferase 1 (SAT1) that facilitates polyamine catabolism and peroxide generation. Loss of SAT1 expression can partially eliminate p53-mediated ferroptosis [64]. Interestingly, although both are p53-induced ferroptosis, p53-mediated SAT1-induced ferroptosis depends on the expression of ALOX15 compared to p53 indirectly releasing ALOX12 from the inactivated ALOX12-SLC7A11 complex, although ALOX15 and ALOX12 belong to the same mammalian lipoxygenase family. Similarly, neither of them is dependent on the regulation of SLC7A11 and GPX4. Recent studies have also shown that iron can also be involved in lipid peroxidation during ferroptosis as a cofactor of POR. POR can increase the peroxidation of PUFAs to promote ferroptosis, mainly by acting in conjunction with NADH-cytochrome b5 reductase (CYB5R1), which uses NADPH and O_2_ as substrates to produce H_2_O_2_ and then participates in the iron-mediated Fenton reaction to cause lipid peroxide accumulation, thereby promoting ferroptosis [65].

Iron effectively works due to its redox potentiality to switch between the two states of ferric iron (Fe^3+^) and ferrous iron (Fe^2+^), and only intracellular free Fe^2+^ (rather than Fe^3+^) induces ferroptosis. Iron could be transported into cells through the transferrin/transferrin receptor (Tf-TfR) system [66]. Cellular iron is mainly stored in ferritin, which is composed of 24 subunits of two types of FTL and FTH1 [67]. Once ferritin binds to its cargo receptor nuclear receptor coactivator 4 (NCOA4), it will be transported to lysosomes for autophagic degradation (ferritinophagy) [30]. Excess trivalent iron is stored in ferritin, whereas ferritinophagy releases iron stored in ferritin into the labile iron pool, thereby regulating iron homeostasis, which determines the sensitivity to ferroptosis [47,68]. Solute Carrier Family 40 Member 1 (SLC40A1, also known as FPN1) is responsible for the exportation of intracellular free Fe^2+^ and the reoxidation of Fe^2+^ to Fe^3+^. Both ceruloplasmin (CP) and hephaestin (HEPH) can oxidize Fe^2+^ and promote FPN1 to export iron, so FPN1/CP and FPN1/HEPH are the main iron efflux pathways [69]. Other recently discovered mechanisms that control intracellular iron abundance affect the sensitivity of cells to ferroptosis. Prominin 2 (PROM2) has also been shown to export intracellular iron by promoting the formation of iron-containing multivesicular bodies (MVBs) and exosomes, thereby inhibiting ferroptosis [70].

ROS. ROS and reactive nitrogen species (RNS) are considered to be important signals of ferroptosis. ROS, including superoxide anion (O_2_•−), hydroxyl radical (•OH), hydrogen peroxide (H_2_O_2_), and singlet oxygen (1O2), are produced endogenously in the normal oxidation process related to cell metabolism [71]. The main sources of cellular ROS production encompass the mitochondrial electron transport chain (mETC) and NADPH oxidase (NOX) on the cell membrane [72]. The NOX family consists of NOX1, cytochrome B-245β chain (CYBB/NOX2), NOX3, NOX4, NOX5, double oxidase 1 (DUOX1), and DUOX2, which are involved in a membrane-bound enzyme complex that, together with other proteins, transport electrons through the plasma membrane to produce superoxide and other downstream ROS [73]. Previous studies have shown that ROS accumulation is considered to be an important indicator of ferroptosis.

Lipid oxidation. Another extremely important indicator of ferroptosis is unrestricted lipid peroxides. Lipid peroxidation primarily involves two categories: enzymatic lipid peroxidation and non-enzymatic lipid peroxidation [74]. Non-enzymatic lipid peroxidation is a chain reaction driven by free radicals. PUFAs are essential for membrane lipid peroxidation, and the initiation of lipid peroxidation requires the removal of a bisallylic hydrogen atom (located between two carbon–carbon double bonds) from the polyunsaturated fatty acyl of the phospholipids (PUFA-PLs) integrated into the lipid bilayer. This leads to the formation of a carbon-centered phospholipid free radical (PL•), which then reacts with molecular oxygen to produce a phospholipid peroxide (PLOO•) and removes hydrogen from another PUFA to form PLOOH [39]. Impaired GPX4 will hinder PLOOH conversion to the corresponding alcohol (PLOH), and the oxidation of PUFAs in the membrane may lead to the spread of the oxidation reaction in the membrane, which will eventually lead to the formation of lipid peroxides such as 4-hydroxynonenal and malondialdehyde [75]. Excessive uncontrolled membrane lipid peroxides can lead to organelle dysfunction and loss of plasma membrane integrity, causing a loss of permeability and leading to cell death via ferroptosis [76,77]. Enzymatic lipid peroxidation is mainly catalyzed by three key enzymes: long-chain acyl-CoA synthetase 4 (ACSL4), lysophosphatidylcholine acyltransferase 3 (LPCAT3), and ALOX. ACSL4 catalyzes the conversion of free PUFAs into acyl-CoA derivatives (PUFA-CoAs), which can be further catalyzed by LPCAT3 to produce PL-PUFAs. Finally, ALOX catalyzes PL to form LOOH to promote ferroptosis [78]. In addition, POR, formerly known as cytochrome c reductase, is an essential membrane-bound flavin protein that transfers electrons to all cytochrome P450 (CYP450) enzymes [79]. POR can promote lipid peroxidation by accelerating the cycling between Fe^2+^ and Fe^3+^ in the CYP450 heme fraction and subsequently ferroptosis [70]. Lipid droplets produced by the endoplasmic reticulum can store lipids in cells and provide lipids for cell metabolism. Lipid droplet cargo receptor RAB7A can mediate selective autophagy (lipophagy) to degrade lipid droplets, increasing the production of free fatty acids and promoting lipid peroxidation [80,81]. As the accumulation of lipid peroxides triggers ferroptosis, lipid metabolism is tightly intertwined with ferroptosis.

## 4. Ferroptosis in the Progression of Pancreatic Cancer

**Figure 3:**
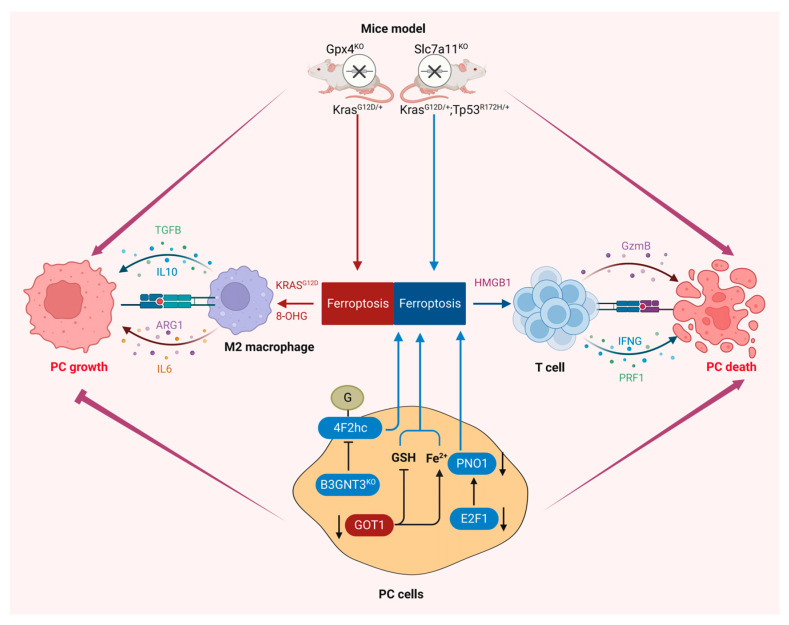
Ferroptosis in the progression of pancreatic cancer. Ferroptosis plays a dual role in pancreatic cancer: On the one hand, ferroptotic PDAC cells in KC mice with the pancreatic knockout Gpx4 will release 8-OHG or KRASG12D to promote the polarization of M2 macrophages and induce the release of related cytokines (e.g., IL6, IL10, ARG1, TGFB), thereby promoting the growth of pancreatic tumors. On the other hand, Slc7a11 knockout KPC mice not only inhibited the synthesis of CoA and GSH to induce ferroptosis, but subsequent ferroptosis cells also activated antigen-specific adaptive immune responses to inhibit tumors. B3GNT3 in PDAC cells inhibits ferroptosis to promote the growth of pancreatic tumors by mediating the protein stability of 4F2hc and enhancing the interaction between 4F2hc and xCT. The inhibition of GOT1 can promote ferroptosis through the enhancement of mitochondrial stress and the dysfunction of redox homeostasis. E2F1 inhibits ferroptosis and promotes malignant progression of pancreatic cancer by the transcriptional activation of PNO1. Created with BioRender.com.

Studies have shown that intervention in the process of ferroptosis will affect the growth and proliferation of pancreatic cancer cells and the progression of pancreatic cancer (Figure 3). The transition of a normal pancreatic duct to pre-invasive precursor lesions into invasive PDAC is caused by multiple factors, but this progress is mainly driven by the intrinsic Kras mutation in the inflammatory tumor microenvironment [82]. Ferroptosis was initially described as a form of Ras-mutation-dependent regulatory cell death with the characteristics of excessive iron-induced oxidative damage [29]. Dai et al. found that ferroptosis can promote the tumorigenesis of pancreatic cancer in pancreatic Gpx4 conditional knockout KC (*Pdx1-Cre*; *kras^G12D/+^*, termed KC mice) or a high-iron diet model. Mechanistically, high-iron diets or Gpx4 depletion led to the release of the oxidative DNA damage product 8-OHG, which activated the TMEM173/cGAS-dependent DNA sensor pathway, which promotes macrophage infiltration and activation during pancreatic tumorigenesis [38]. Autophagy, as the main mechanism mediating the degradation and recycling of various cell cargos to lysosomes [83], has been reported to play an important role in the occurrence, development, and drug resistance of pancreatic cancer [84,85,86]. Autophagy-dependent ferroptosis can release KRAS^G12D^ protein from PDAC cells into exosomes in an autophagy-dependent manner, leading to macrophage polarization into a tumor M2 phenotype. The inhibition of KRAS^G12D^ release by tumor cells and uptake by immune cells in vivo can reverse macrophage-induced PDAC tumor growth [35].

As an interesting finding in a genetically engineered mouse (*Kras^FSF.G12D/+^*; *Tp53^R172H/+^*; *Pdx1FlpO^tg/+^*; *Slc7a11^Fl/Fl^*; *Rosa26^CreERT2/+^*, termed KPFSR mice) model, in which the administration of tamoxifen induces systemic deletion of Slc7a11, KPFSR mice receiving tamoxifen exhibited the induction of tumor-selective ferroptosis and inhibition of pancreatic cancer growth, and the administration of cyst(e)inase can replicate the result in KPC mice [87]. Studies in two mouse models of pancreatic cancer have shown the opposite role of ferroptosis in pancreatic cancer [35,38]. It is undeniable that the state of TP53 is an important reason for its opposite results, but the functional mechanisms of ferroptosis-dependent and non-ferroptosis-dependent Gpx4 and Slc7a11 in pancreatic cancer are also worthy of further elucidation. The inhibition of cytosolic aspartate aminotransferase (GOT1) represses mitochondrial metabolism and promotes a catabolic state, which enhances the availability of unstable iron through autophagy, triggering ferroptosis and subsequent pancreatic cancer cell death [88]. One of the important reasons for the high malignancy of pancreatic cancer is the acquired change of abnormal protein glycosylation, which pathologically reshapes the molecular biological process and protects pancreatic cancer cells from death. B3GNT3-mediated glycosylation of 4F2hc can promote its protein stability and enhance the interaction between 4F2hc and xCT, thereby protecting pancreatic cancer cells from ferroptosis [89]. The RNA-binding protein partner of NOB1 (PNO1) is a ribosome assembly factor that is essential for ribosome biogenesis. Previous results have shown that silencing PNO1 in pancreatic cancer shows a series of characteristics of ferroptosis activation. Mechanistically, E2F Transcription Factor 1 (E2F1) may bind to the promoter of PNO1 to promote the transcriptional expression of PNO1, thus inhibiting ferroptosis and promoting the malignant progression of pancreatic cancer [90]. However, whether the process of pancreatic cancer promoted by PNO1 is ferroptosis-dependent remains to be discussed.

Cancer cells can inhibit immune responses directly or through other cells in the tumor microenvironment to promote survival and tumor formation. Inhibition of T-cell checkpoint-related proteins has shown great promise in a variety of cancer types. The simplest form of immunotherapy is a cancer vaccine using tumor-specific antigens. Unfortunately, pancreatic cancer is not sensitive to the use of these drugs [91]. Recent studies have found that ferroptosis in pancreatic cancer may be closely related to immunotherapy.

M2 tumor-associated macrophages (TAMs) can express immunosuppressive signals to promote the proliferation and invasion of pancreatic cancer cells [92,93]. The release of KRAS^G12D^ protein from ferroptotic PDAC cells through autophagy-mediated secretion in exosomes results in macrophage polarization to the tumor M2 phenotype [38]. In pancreatic cancer cells, proteoglycan decorin (DCN) released by ferroptosis triggers innate and adaptive immune responses. DCN is released and binds to advanced glycosylation end-product-specific receptor (AGER) on macrophages. The production of pro-inflammatory cytokines is triggered in an NF-κb-dependent manner to enhance anti-tumor immunity [94]. An early study also found that High Mobility Group Box 1 (HMGB1) released by ferroptosis cells in an autophagy-dependent manner can also enhance anti-tumor immunity. HMGB1 is a damage-associated molecular pattern (DAMP) molecule. A DAMP is an endogenous danger signal that alerts the innate immune system and shapes the inflammatory response after cell death [95]. In general, current research on ferroptosis and pancreatic cancer treatment is still limited. Although some bioinformatics-based studies have shown that ferroptosis regulators are associated with immune cell infiltration in pancreatic cancer, further experimental verification is needed [96,97,98]. Research on ferroptosis and immunotherapy will provide new research ideas for the treatment and prevention of pancreatic cancer.

## 5. Ferroptosis in the Treatment of Pancreatic Cancer

**Figure 4:**
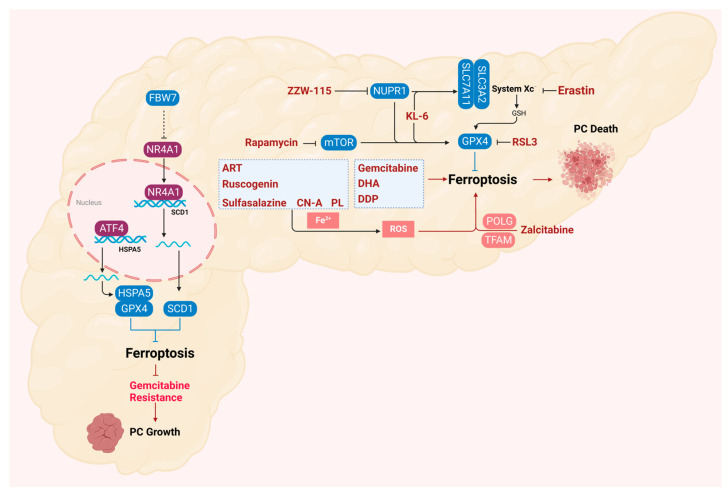
Drug-induced ferroptosis in pancreatic cancer cells. ATF4 inhibits ferroptosis and increases gemcitabine resistance by promoting the transcriptional expression of HSPA5, thereby promoting the stability of GPX4. Low expression of FBW7 in tumors relieves its inhibition of NR4A1, thereby promoting the expression of SCD1 to inhibit ferroptosis and promote gemcitabine resistance. The anticancer and procancer effects of various drugs have been shown to be achieved by inducing/inhibiting ferroptosis. Created with BioRender.com.

Currently, the efficacy and outcome of pancreatic cancer treatment largely depend on the stage of the disease at the time of diagnosis, but less than 20% of pancreatic cancer patients have resectable tumors [99]. Following upfront resection, adjuvant chemotherapy has been routinely used to prevent early tumor recurrence [100]. The limited clinical benefit of anticancer drugs arises from the fact that tumor cells evade drug-induced cell death, especially apoptosis [91,101]. Therefore, the development of new drugs or effective combination therapies is imminent, and the induction of lipid-peroxidation-mediated ferroptosis may offer new insights and potential therapeutic strategies for overcoming apoptosis resistance and inducing pancreatic cancer cell death (Figure 4).

Small molecules that activate ferroptosis through endogenous pathways are important signaling pathway regulators. In pancreatic cancer cells, the interaction between rapamycin kinase target protein (mTOR) and GPX4 signaling plays an important role in regulating autophagy-dependent ferroptosis. The classical autophagy inducer rapamycin and the classical ferroptosis activator RSL3 can block the activation of mTOR in human pancreatic cancer cells and cause the degradation of the GPX4 protein [102]. Zalcitabine is an antiviral drug used to treat human immunodeficiency virus infection and induces mitochondrial DNA stress, which activates the STING1/TMEM173-mediated DNA sensing pathway, leading to autophagy-dependent ferroptosis, thereby inhibiting the growth of primary and immortalized human pancreatic cancer cells [103]. There is evidence that ZZW-115, one of the most famous inhibitors of NUPR1, induces mitochondrial-dependent ferroptosis in MiaPaCa-2 cells by inhibiting the expression of GPX4 and SLC7A11 [104]. Studies have demonstrated that KL-6, a derivative of benzochalcone, can effectively inhibit the growth of pancreatic cancer cells in a dose-dependent manner. Additionally, KL-6 can regulate the expression of related pathway genes in various cancers, including ferroptosis [105].

Naturally derived ferroptosis-inducing drugs have also been reported to have excellent effects on pancreatic cancer. Piperlongumine (PL), a natural product, induces cancer cell death by significantly increasing intracellular ROS levels and at least partly by inducing ferroptosis, and cancer-cell-killing activity can be reversed by ferroptosis inhibitors. Cotylenin A (a plant growth regulator) and PL synergistically induce pancreatic cancer cell death, and ferroptosis inhibitors and DFO can eliminate this combined effect. Sulfasalazine (clinically approved) combined with PL and CN-A triple therapy is more effective for pancreatic cancer [106]. Studies have shown that ruscogenin significantly inhibited the viability of pancreatic cancer cells and induced cell death in a dose- and time-dependent manner [107]. The natural compound artesunate (ART) is an antimalarial drug worthy of attention. Previous studies have shown that ART is a specific inducer of ferroptosis in many different types of cancers, including pancreatic cancer. ART showed higher cytotoxicity in Ras-mutant PDAC cells than in wild-type Kras-expressing PDAC cells [108]. It is worth noting that although the underlying molecular mechanism is still unclear, some studies have found that ART mainly regulates the presence of intracellular iron to induce ferroptosis [109,110,111].

## 6. Ferroptosis in the Drug Resistance of Pancreatic Cancer

For patients with locally advanced or metastatic pancreatic cancer, chemotherapy plays a vital role in the treatment of pancreatic cancer [100]. Gemcitabine, as a first-line drug, can be used alone or in combination for the treatment of patients with advanced pancreatic cancer. For resectable tumors, surgery followed by adjuvant chemotherapy (gemcitabine plus capecitabine) is the standard treatment. In metastatic pancreatic cancer, folic acid and albumin–paclitaxel–gemcitabine are the standard of care for patients with good performance, but the overall remission rate of the above methods is limited [91]. Exploring the mechanism of forms of non-apoptotic cell death, such as ferroptosis, provides an opportunity to overcome chemotherapy-induced apoptosis resistance in PDAC (Figure 4).

Ferroptosis inducers erastin and RSL3 have been reported to increase the killing effect of gemcitabine on pancreatic cancer cells [112,113]. Gemcitabine induces the expression of endoplasmic-reticulum-stress-related transcription factor 4 (ATF4). Subsequently, Heat shock 70-kDa protein 5 (HSPA5), a switch factor in the endoplasmic reticulum stress response, is upregulated by ATF4-dependent transcriptional regulation and then binds to GPX4 to protect GPX4 protein degradation, thereby limiting the anticancer activity of gemcitabine [114].

F-box and WD repeat domain-containing 7 (FBW7) is a substrate recognition component of the Skp1-Cul1-F-box (SCF) ubiquitin ligase complex, which can exert tumor inhibition by targeting oncoprotein degradation. FBW7 inhibits the expression of stearoyl-CoA desaturase (SCD1) by inhibiting nuclear receptor subfamily 4A group member 1 (NR4A1), thereby promoting ferroptosis and apoptosis [113]. Dihydroartemisinin (DHA) is a safe and promising therapeutic drug that can induce ferroptosis in cancer cells [115]. Studies have shown that DHA can enhance the cytotoxicity of cisplatin (DDP) in vitro and in vivo. Ferroptosis induced by DHA and DDP promotes the cytotoxicity of the combination therapy, inhibits the proliferation of PDAC cells, and induces DNA damage [115,116]. Experiments in vitro have shown that gemcitabine combined with cisplatin can induce ferroptosis in AsPC1 cells, and the mechanism may be related to the upregulation of SAT1 expression [117]. The above results indicate that the combination of ferroptosis inducer therapy will provide a feasible strategy to overcome the chemoresistance of PDAC.

## 7. Ferroptosis in Pancreatitis

**Figure 5:**
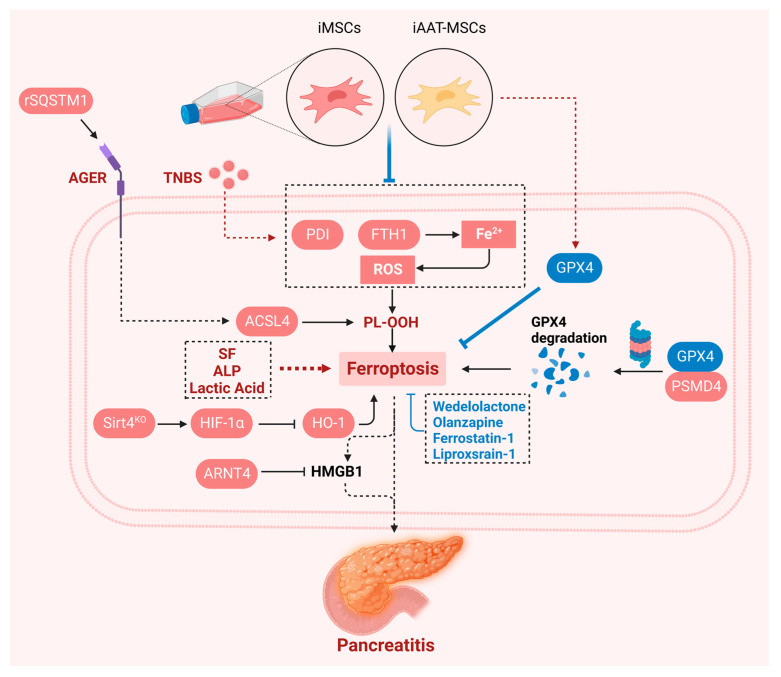
Ferroptosis in pancreatitis. A large number of epidemiological studies have shown that pancreatitis is an important risk factor for pancreatic cancer. Recombinant SQSTM1 protein increases the production of PUFAs through the AGER-ACSL4 axis, leading to the occurrence of autophagy-dependent ferroptosis, thereby mediating the progression of AP. iMSCs and iAAT-MSCs ameliorate ferroptosis in acinar cells by regulating the FTH1-PDI-GPX4 axis, regulating ROS function and iron production. ARNTL prevents experimental acute pancreatitis by blocking the ferroptosis-mediated release of HMGB1. PSMD4 promotes GPX4 degradation to promote ferroptosis and pancreatic inflammation. Created with BioRender.com.

Pancreatitis, an inflammatory disorder of the pancreas, is associated with substantial morbidity and with limited treatment options [118,119]. Pancreatitis is a risk factor for pancreatic cancer. In patients with unexplained pancreatitis, chronic pancreatitis, and hereditary pancreatitis, the Relative Risk of pancreatic cancer is 5.1 (95%CI: 3.5–7.3), 13.3 (95%CI: 6.1–28.9), and 69 (95%CI: 56.4–84.4), respectively [120]. Recent studies have shown that ferroptosis also plays an important role in the occurrence of pancreatitis (Figure 5). The circadian transcription factor ARNTL (also known as BMAL1) prevents experimental acute pancreatitis by blocking the ferroptosis-mediated release of HMGB1 (a sterile inflammatory mediator). In contrast, in Arntl-deficient mice, lipstatin-1 (a ferroptosis inhibitor) or anti-HMGB1 neutralizing antibody alleviated the development of acute pancreatitis [121]. Trypsin is a serine protease secreted by pancreatic acinar cells. Studies have shown that trypsin-induced PSMD4-dependent GPX4 degradation plays a role in the sensitivity of acinar cells to ferroptosis. It was further demonstrated that in experimental models of acute or chronic pancreatitis, olanzapine inhibited ferroptosis in pancreatic Gpx4 KO mice, improved pancreatic function, reduced inflammation, and limited tissue damage [122]. An analysis based on clinical datasets also showed that ferroptosis-related factors (such as serum iron, ALP, lactic acid, etc.) are independent factors affecting mortality and prognosis in patients with acute pancreatitis [123]. Similarly, another result also shows the complex functions and important roles of ferroptosis-related genes in acute pancreatitis, but its specific potential mechanisms still need to be further explored [124]. Acute kidney injury (AKI) is a common complication of severe acute pancreatitis (SAP). Studies have shown that inhibition of ferroptosis can alleviate renal lipid peroxidation during acute pancreatitis and reduce pathological damage to the pancreas and kidney tissue [125]. Another study also supported the above results, and the increased production of oxidation products and lipid peroxidation markers in Sirt4 KO mice indicated that Sirt4 was involved in inflammation and oxidative stress during SAP. Further studies have shown that Sirt4 plays a protective role in SAP. Deletion or overexpression of Sirt4 affects the expression level of hypoxia-inducible factor-1α (HIF-1α) after SAP induction and regulates the expression of ferroptosis-related proteins by mediating the HIF-1α/HO-1 pathway [126]. The natural compound Wedelolactone has strong anti-inflammatory and antioxidant activities. Studies have shown that ferroptosis plays a crucial role in AP. In the mouse AP model induced by taurocholic acid or caerulein, the ferroptosis inhibitor ferrostatin-1 significantly reduced AP and its associated lung injury. In vitro and in vivo data showed that oxidative stress and lipid peroxidation were also inhibited in the Wedelolactone-treated group, and the expression of the ferroptosis antagonistic marker GPX4 was upregulated [127]. Patients with hereditary iron overload will have a large amount of iron deposition in the pancreas. In a mouse model, it was also found that iron-overload-induced chronic pancreatitis, accompanied by increased oxidative stress in the pancreas, increased malondialdehyde levels and decreased SOD and glutathione peroxidase activities [128]. Dedifferentiation of acinar cells is one of the most prominent features of acute and chronic pancreatitis. Studies have shown that SLC7A11 specifically prevents ferroptosis of acinar cells by supplementing glutathione pools and maintaining ROS balance. This also suggests that SLC7A11 is essential for the fate of acinar cells under stress conditions [129]. In an in vitro cell culture system, the protective effect of immortalized human mesenchymal stem cells (iMSCs) and overexpression of α-1 antitrypsin (iAAT-MSCs) on 2,4,6-trinitrobenzene sulfonic acid (TNBS)-induced acinar cell death was shown. Further mechanistic studies showed that iMSCs and iAAT-MSCs improved ferroptosis in acinar cells by regulating the FTH1/protein disulfide isomerase (PDI)/GPX4 signaling pathway and regulating ROS function and iron production [130]. Previous studies have shown that autophagy is essential for ferroptosis [30,68]. Extracellular SQSTM1 mediates AP by enhancing sensitivity to autophagy-dependent ferroptosis. Mechanically, the recombinant SQSTM1 protein increases the expression of AGER-dependent ACSL4, leading to the production of PUFAs, resulting in the formation of autophagosomes and subsequent ferroptosis [131]. In summary, ferroptosis plays an important role in both acute and chronic pancreatitis. Further revealing the mechanism of ferroptosis in different types of pancreatitis is particularly important for the determination of the progression of pancreatitis, the evaluation of targeted therapy and the prevention and treatment of pancreatitis-induced pancreatic cancer.

## 8. Ferroptosis in Insulin Resistance

**Figure 6:**
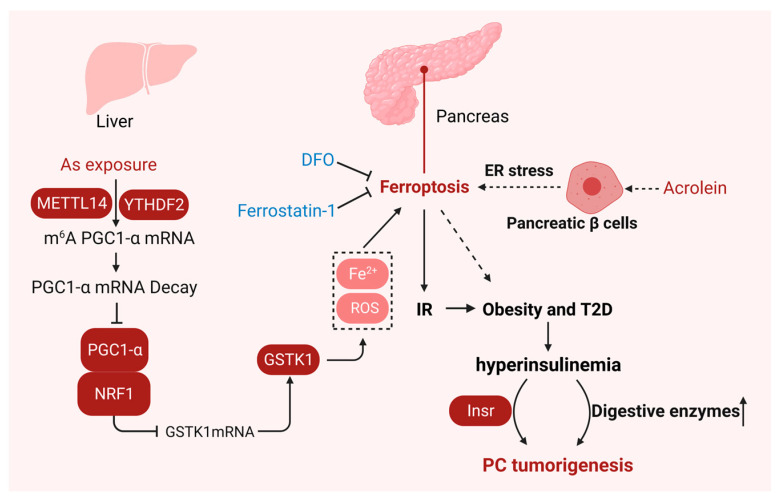
Ferroptosis in insulin resistance. Ferroptosis is essential for the occurrence of insulin resistance. METTL14- and YTHDF2-mediated m6A modification of PGC-1α promotes NRF1/GSTK1-dependent ferroptosis and hepatic insulin resistance. Ferroptosis can induce islet dysfunction, and ferroptosis inhibitors such as Fer-1 and DFO can reverse this process. Created with BioRender.com.

Insulin, a hormone produced by pancreatic β cells, is mainly responsible for stimulating glucose uptake and storage to regulate blood glucose levels [132]. Insulin resistance is clinically defined as the biological effect of a given concentration of insulin that is lower than expected [133]. Recent studies have shown that ferroptosis also plays an important role in the occurrence of insulin resistance (Figure 6).

Studies have shown that ferroptosis is an important process that mediates the pathogenesis and progression of T2DM, and insulin resistance can be induced in the liver, fat, and muscle by ferroptosis [134]. Pancreatic β-cell dysfunction leads to insulin insensitivity in target tissues and accelerates the development of insulin resistance [135]. Previous studies in vitro have shown that islets are indeed susceptible to ferroptosis, and the induction of ferroptosis can lead to impaired islet function. When treated with erastin, the glucose-stimulated insulin secretion (GSIS) capacity of pancreatic β cells was significantly reduced in vitro. In contrast, GSIS-induced damage was reversed upon pretreatment with ferroptosis inhibitors Fer-1 or DFO [136]. Acrolein is a typical food and environmental pollutant and a risk factor for diabetes. Studies have shown that acrolein causes islet β-cell damage and insulin secretion disorder through endoplasmic-reticulum-stress-induced ferroptosis. Acrolein treatment induces the PERK-eIF2α-ATF4 branch in the endoplasmic reticulum stress response to promote the expression of transcription factor CHOP, while high expression of CHOP inhibits PPARγ-mediated GSH production and induces ferroptosis [137]. Similarly exciting studies have shown that arsenic exposure is closely related to hepatic insulin resistance (IR) and ferroptosis. Sodium arsenite (NaAsO_2_) inhibits PGC-1α protein expression through METTL14- and YTHDF2-mediated m6A methylation, promoting NRF1/GSTK1-dependent ferroptosis and hepatic insulin resistance. Inhibition of ferroptosis can reverse the decrease in hepatic insulin sensitivity caused by NaAsO_2_ [138]. The above studies have shown that ferroptosis plays an important role in the occurrence of insulin resistance. Although epidemiological studies have shown that insulin resistance significantly increases the risk of pancreatic cancer [139], the specific mechanism of insulin resistance in the progression of pancreatic cancer is poorly understood. But a recent exciting study has brought breakthroughs. Insulin resistance is thought to lead to hyperglycemia, which then drives pancreatic β cells to secrete more insulin to maintain glucose homeostasis, leading to hyperinsulinemia [140]. Obesity and T2DM are risk factors for pancreatic cancer [141]. Their association with cancer may be partly related to elevated levels of insulin-like growth factor-1 (IGF-I) and hyperinsulinemia secondary to insulin resistance [142]. High levels of insulin in patients with hyperinsulinemia activate insulin/IGF signal transduction, which in turn activates phosphatidylinositol 3-kinase (PI3K)/Akt/mTOR and mitogen-activated protein kinase (MAPK) signaling pathways to promote cancer cell growth, survival, motility, and drug resistance [133]. Epidemiological studies have shown that hyperinsulinemia is associated with an increased risk of PDAC and poor survival rates, while reducing insulin production can inhibit pancreatic intraepithelial neoplasia (PanIN) precancerous lesions in Kras mutant mice [140,143]. A recent study showed that the insulin receptor (Insr) in pancreatic acinar cells of Kras^G12D^ is essential for the formation of hyperinsulinemia-driven PanIN under the condition of high-fat-diet-induced obesity, and its loss can inhibit the occurrence of hyperinsulinemia-associated pancreatic cancer, revealing the mechanism of the link between hyperinsulinemia and pancreatic cancer [144]. Therefore, whether ferroptosis affects the progression of pancreatic cancer through insulin resistance will also be an interesting concern in the future. We summarized the gene regulators and agents involved in ferroptosis in the progression and treatment of pancreatic-related diseases as shown in Table 1.

## 9. Conclusions

It is worth noting that the existing results show that the knockout of Slc7a11 and Gpx4 in distinct mouse models of pancreatic cancer have different results in the process of pancreatic cancer [35,38,87]. This gives us an important hint that when targeting ferroptosis to treat pancreatic cancer, strategies must be developed with full consideration of the patient’s genetic context. In addition, ferroptosis is also an autophagy-dependent form of cell death, and overactivation of selective autophagy promotes ferroptosis [68,156]; the impairment of autophagy degradation has also been reported to promote or inhibit Kras^G12D^-induced pancreatic tumorigenesis according to the state of TP53 in mice [157]. The double-edged sword role of ferroptosis cannot be ignored when making judgments concerning the treatment of pancreatic cancer; simply promoting and inhibiting ferroptosis cannot limit the progression of pancreatic cancer.

Most studies have shown that pancreatic cancer is not sensitive to immunotherapy [100]. The discovery of ferroptosis may delineate new avenues for immunotherapy of pancreatic cancer [38]. Regrettably, the reported studies on ferroptosis and immune cell infiltration in pancreatic cancer are mainly focused on big data analysis, and in-depth examination of the in vitro and in vivo studies and molecular mechanisms is still lacking, which greatly limits our understanding of the treatment and prevention of pancreatic cancer based on its immune characteristics through ferroptosis.

In the past decade, chemotherapeutic drugs that trigger ferroptosis and enhance the inhibitory effect of chemotherapeutic drugs on a variety of tumors, including pancreatic cancer, have gradually been found [158,159]. However, the practical application of these treatment methods faces many challenges. An important problem to be solved is how to avoid the occurrence or aggravation of pathological conditions, such as neurodegenerative diseases or ischemic diseases caused by ferroptosis, while exerting anticancer activity [160]. A feasible strategy is to elucidate the molecular mechanism of ferroptosis induced by existing clinical drugs, such as gemcitabine, sorafenib, etc. [114,161], in addition to the classical anticancer activity, and to amplify their ferroptosis induction in drug-resistant tumor cells.

Pancreas-related diseases including pancreatitis, obesity, and T2DM have been reported to be significantly associated with the risk of pancreatic cancer [120,141]. Although the conclusion based on published epidemiological studies has been reported, the underlying molecular mechanism is still unclear. This review also summarizes some of the state-of-the-art advances in the occurrence of ferroptosis in pancreatitis- and insulin-resistance-associated pancreatic cancer. Given the important role of ferroptosis in pancreatitis and insulin resistance, the mechanism of ferroptosis in pancreatitis- and insulin-resistance-associated pancreatic cancer will provide a new perspective for elucidating the tumor onset of pancreatic cancer.

## Figures and Tables

**Table 1:** List studies on ferroptosis-related gene regulators or agents in pancreatic cancer or pancreatitis.

Gene Regulators						
Name	Mechanism or Target	Driver or Suppressor	Study Type	Outcomes	Indication	References
GPX4	Targeting lipid metabolism	Suppressor	KC mice	High-iron diets or Gpx4 depletion promoted 8-OHG release and thus activated the TMEM173/STING pathway and subsequent macrophage infiltration Kras-driven PDAC in mice	Pancreatic cancer	[38]
KRAS	Targeting lipid metabolism	Driver	KRAS^G12D^ mice	KRAS^G12D^ promoted macrophages to switch to M2-like phenotype via STAT3-dependent fatty acid oxidation	Pancreatic cancer	[35]
SLC7A11	Targeting oxidative stress	Suppressor	KPC mice	The import of oxidized cysteine (cystine) via system x_C_^−^ is a critical dependency of pancreatic cancer	Pancreatic cancer	[87]
GOT1	Targeting iron homeostasis	Suppressor	PDAC cell lines	GOT1 inhibition represses mitochondrial metabolism and promotes a catabolic state	Pancreatic cancer	[88]
B3GNT3	Targeting oxidative stress	Suppressor	PDAC cell lines	B3GNT3 catalyzes the glycosylation of 4F2hc, stabilizes the 4F2hc protein, and enhances the interaction between 4F2hc and xCT	Pancreatic cancer	[89]
PNO1	Unclassified	Suppressor	PDAC cell lines	Knockdown of PNO1 promotes ferroptosis in PANC-1 cells	Pancreatic cancer	[90]
DCN	Unclassified	Unclassified	PDAC cell lines, HT1080, HeLa	DCN released by ferroptotic cells activates AGER-dependent tumor-protective immune response	Pancreatic cancer, etc.	[94]
HMGB1	Unclassified	Unclassified	PDAC cell lines, HT1080	HMGB1 released by ferroptotic cells activates AGER-dependent inflammation in macrophages	Pancreatic cancer, etc.	[95]
RBCK1	Targeting oxidative stress	Suppressor	PDAC cell lines	RBCK1 promotes proteasome degradation of MFN2, resulting in reduced ROS production and lipid peroxidation	Pancreatic cancer	[145]
FOSL1	Targeting oxidative stress	Driver	PDAC cell lines	KRAS/FOSL1/TFRC axis promoted the PDAC cells vulnerable to alteration of the iron level in the tumor microenvironment	Pancreatic cancer	[146]
OSGIN1	Targeting oxidative stress	Suppressor	PDAC cell lines	OSGIN1 directly enhanced GCLM activity	Pancreatic cancer	[147]
TMOD3	Targeting lipid metabolism	Driver	PDAC cell lines, BALB/c nude mice and C57BL/6 mice	TMOD3 facilitated autophagic degradation of ACSL4	Pancreatic cancer	[148]
IL15	Targeting lipid metabolism	Suppressor	PANC-1 and SW1990 cell lines, BALB/c nude mice	IL15 activates IL15RA-STAT3 axis to promote GPX4 and ACSL3	Pancreatic cancer	[149]
circ_0005397	Targeting oxidative stress	Suppressor	PDAC cell lines	Circ_0005397 promotes PCBP2 expression through KAT6 A and H3K9 ac	Pancreatic cancer	[150]
PRMT6	Targeting oxidative stress	Suppressor	PDAC cell lines	PRMT6 mediates the asymmetric dimethylarginine modification of p62 to facilitate its phase separation, preventing Keap1 from activating Nrf2 signaling and inhibiting ferroptosis	Pancreatic cancer	[151]
ARID3A	Targeting lipid metabolism	Suppressor	PDAC cell lines	The inhibition of ARID3A attenuates transcriptional repression of PTEN, leading to GPX4 depletion and increased lipid peroxidation	Pancreatic cancer progress and chemosensitivity	[152]
FBW7	Targeting lipid metabolism	Driver	PDAC cell lines	FBW7 inhibits the expression of SCD1 by inhibiting NR4A1	Chemosensitivity	[113]
ELAVL1	Targeting oxidative stress	Driver	Rat pancreatic exocrine cells	ELAVL1-dependent SOAT2 exacerbated pancreatic exocrine cell injury	Pancreatitis	[153]
ARNTL	Targeting oxidative stress	Suppressor	Acute pancreatitis model mice induced by l-arginine	ARNTL prevents experimental acute pancreatitis by blocking ferroptosis-mediated release of HMGB1	Acute pancreatitis	[121]
SIRT4	Targeting oxidative stress		Acute pancreatitis model mice induced by l-arginine	SIRT4 regulates the expression of ferroptosis-related proteins by mediating HIF-1α/HO-1 pathway	Acute pancreatitis	[126]
SQSTM1	Targeting lipid metabolism	Driver	Acute pancreatitis model mice induced by caerulein	Recombinant SQSTM1 protein increases the expression of AGER-dependent ACSL4	Acute pancreatitis	[131]
C1QTNF5	Targeting iron homeostasis	Driver	SAP mouse model through pancreatic duct ligation (PDL)	Myonectin promoted iron-accumulation-induced ferroptosis leading to acinar cell necrosis	Severe acute pancreatitis	[154]
**Agents**						
**Name**	**Mechanism or Target**	**Driver or Suppressor**	**Study type**	**Outcomes**	**Indication**	**References**
Gemcitabine		Driver	PDAC cell lines	Gemcitabine time-dependently increased GPX4 protein expression and GPX4 activity	Chemosensitivity	[114]
Zalcitabine	Targeting lipid metabolism	Driver	PDAC cell lines	Zalcitabine induced mitochondrial DNA stress and contributed to macroautophagy/autophagy-dependent ferroptotic cell death via lipid peroxidation	Pancreatic cancer	[103]
ZZW-115	Targeting iron homeostasis	Suppressor	PDAC cell lines, knockout mice	ZZW-115 inhibited completely the translocation of NUPR1 from the cytoplasm to the nucleus by competing with importins	Pancreatic cancer	[104]
KL-6	Unclassified	Unclassified	PDAC cell lines	KL-6 promoted lipid oxidation in a dose-dependent manner	Pancreatic cancer	[105]
Piperlongumine/Cotylenin A/Sulfasalazine	Unclassified	Unclassified	PDAC cell lines	Piperlongumine induced ROS production	Pancreatic cancer	[106]
Ruscogenin	Targeting iron homeostasis	Driver	PDAC cell lines	Ruscogenin increased intracellular ferrous concentration and ROS production	Pancreatic cancer	[107]
Artesunate	Targeting iron homeostasis	Driver	PDAC cell lines	ART promotes the lysosomal degradation of ferritin	Pancreatic cancer	[108]
DHA	Targeting oxidative stress	Driver	PDAC cell lines	Combination therapy with DHA and cisplatin increased mitochondrial-derived ROS accumulation	Chemosensitivity	[116]
Fatostatin	Targeting lipid metabolism	Driver	BxPC-3 and MIAPaCa-2 cell lines, BALB/c nude mice	Fatostatin inhibits SREBP1 transcription-mediated GPX4 upregulation	Pancreatic cancer	[155]
